# A thermal cauterization therapy in dysmenorrhea pain management: A siddha case report

**DOI:** 10.6026/973206300221555

**Published:** 2026-03-31

**Authors:** S Kanimozhi1, R Sathish Adithya

**Affiliations:** 1 Department of Anatomy, Sri Muthukumaran Medical College Hospital and Research Institute, Chennai-69, Tamil Nadu, India; 2 Department of Department of Forensic Medicine and Toxicology (Nanju Maruthuvam), National Institute of Siddha, Chennai-47, Tamil Nadu, India

**Keywords:** Suttigai, Siddha, dysmenorrhea, thermal cauterization, yoga

## Abstract

Dysmenorrhea is a common gynecological condition affecting nearly 70% of adolescent and young adult women in India, often leading to significant physical discomfort and disruption of daily activities. Therefore, it is of interest to investigate the therapeutic efficacy of Thermal cauterization, a traditional Siddha thermal cauterization therapy, administered to a 23-year-old female medical student with recurrent dysmenorrhea unresponsive to NSAIDs. The patient underwent three sessions of thermal cauterization therapy applied to the umbilical region five days prior to menstruation, followed by a six-month regimen of yoga, pranayamam and dietary modifications. Clinical outcomes were assessed using the WaLIDD score and the Dysmenorrhea Symptom Interference (DSI) Scale, revealing substantial improvements in pain intensity, duration, sleep quality, concentration and physical activity; working ability improved from Grade 3 (always affected) to Grade 1 (almost never affected) and pain distribution was completely resolved. Thus, we show that thermal cauterization therapy, when integrated with lifestyle interventions, offers a reproducible and patient-responsive approach for the effective management of dysmenorrhea.

## Background:

Dysmenorrhea, commonly referred to as painful menstruation, is a prevalent gynecological condition characterized by cramping pain in the lower abdomen occurring before or during menstruation [1]. It is broadly classified into two types: primary dysmenorrhea, which occurs in the absence of identifiable pelvic pathology and typically affects adolescents and young women and secondary dysmenorrhea, which is associated with underlying gynecological disorders such as endometriosis, adenomyosis, pelvic inflammatory disease, or uterine fibroids [2]. Despite its ubiquity, dysmenorrhea remains under- recognized as a serious clinical entity [3]. Globally, it affects 45-95% of menstruating women, with 10-25% experiencing symptoms severe enough to disrupt daily functioning. In India, prevalence among adolescent girls exceeds 70%, often leading to absenteeism, reduced academic performance and emotional distress [4]. The causes range from hormonal imbalances and excessive prostaglandin release to uterine hypercontractility and structural abnormalities [5]. General symptoms include lower abdominal pain, backache, nausea, vomiting, diarrhea, fatigue, headache and mood fluctuations [6]. The condition predominantly affects women between 19 to 23 years, though secondary forms may present later in reproductive life [7]. The pain mechanism in primary dysmenorrhea is primarily mediated by excessive prostaglandin F2α which induces uterine ischemia, myometrial hypercontractility and peripheral nerve sensitization [8]. Inflammatory and neuropeptides further exacerbate the pain response [9]. Diagnosis remains largely subjective and symptom-based, often leading to under diagnosis or misclassification. Elevated levels of prostaglandins, leukotrienes and C-reactive protein (CRP) may support diagnosis, but lack specificity [10]. The Siddha system provides a rich repository of external therapies that address pain, inflammation and systemic imbalance without pharmacological burden [11]. Siddha therapy emphasizes the initiation of the body's self healing capacity through external modalities, thereby providing effective relief in musculoskeletal pain without pharmacological dependence [12]. Thermal cauterization (Cauterization Therapy in Siddha Medicine) is a Siddha external therapy categorized specifically under 32 external therapies and exhibits therapeutic potential in the management of various inflammatory conditions. When administered at anatomically defined surface points may elicit systemic symptom relief, offering a non-pharmacological alternative for pain and inflammation control [13]. There are 3 types of Thermal cauterization namely Heated air or gas cauterization, Sun rays cauterization, Thermal cauterization using Ulogam (Cauterization using metal instruments, such as iron rods or plates, heated to a specific temperature) and each type of Thermal cauterization is selected based on the nature of the disease, location of pathology and individual body constitution of the patient [13]. Yoga, with its emphasis on mind-body integration, complements Siddha therapy by reducing stress, enhancing pelvic blood flow and improving hormonal balance [14]. Therefore, it is of interest to report a streamlined, patient-tailored approach that has the potential to transform clinical practice, improve patient outcomes and enrich the evolving landscape of evidence-based traditional medicine.

## Case presentation:

A 23-year-old female medical student arrived at our clinic with menstrual cramps in her calf muscles and excruciating lower abdomen pain. In addition, she reported experiencing modest mood swings, worry, difficulty concentrating and sporadic nausea. According to reports, the pain was severe, recurrent during each menstrual cycle and insensitive to NSAIDs that had previously been provided. For the previous three years, her periods had been terrible. The symptoms, which usually started five hours after the start of her period and lasted for 48 to 72 hours, had a major impact on her academic schedule and frequently required her to take time off or leave college early on the first day of her cycle. She said that her symptoms had gotten worse over the past several months, even though she had previously participated in sports and was in good physical health. Her medical history showed no signs of PCOS, smoking, or systemic illness, no family history of menstrual abnormalities and no history of dysmenorrhea throughout her school years. Menarche occurred at age 13, according to the menstrual history, which also showed normal 28-day cycles, moderate flow during the first three days, dark red flow with clots and bleeding lasting five to six days. Her height of 168 cm, weight of 71.2 kg and BMI of 25.2 from the general examination indicated that she was overweight. There were no anomalies found in the vulva, urethral meatus, or perineal area during the physical examination or pelvic examination. A somewhat low RBC count (3.8x10^12^/L) was found in laboratory tests, but normal levels of haemoglobin (13.5 g/dL), platelets (179 x 109/L), white blood cells (5x10^9^/L) and ESR (7mm/hr) were found. Ultrasonography of the abdomen revealed no structural disease. Based on the lack of corresponding clinical indications, such as pain that persists after menstruation, inter menstrual haemorrhage, dyspareunia, or pelvic enlargement, differential diagnoses such as endometriosis, pelvic inflammatory disease and fibroids were disregarded. The diagnosis of was made as primary dysmenorrheal from above observations.

## Therapeutic intervention:

Following informed consent, the patient was initiated on a therapeutic regimen in which Thermal cauterization served as the primary intervention for dysmenorrhea management.

## Thermal cauterization therapy:

We administered thermal cauterization for this patient. Thermal cauterization therapy was administered using a hammer-shaped iron probe approximately 2 inches in length, applied over a round piece of ginger measuring 1-2 mm in thickness, placed at the umbilicus along with crystal salt as needed. The probe was heated to a red-hot state using a gas stove and carefully positioned over the ginger, allowing the heat to penetrate through the salt and ginger without directly harming the skin. The crystal salt played a crucial role in evenly distributing the thermal energy, acting as a buffer to prevent superficial burns while ensuring effective heat transfer to the underlying tissues (Figure 1 - see PDF). To maintain consistent temperature throughout the procedure, the probe was intermittently reheated and reapplied, sustaining therapeutic warmth for approximately 10 minutes. This intervention was performed as a single sitting, scheduled five days prior to the anticipated onset of menstruation (Table 1 - see PDF).

## Yoga:

As a part of enhancing menstrual health, a structured daily yoga regimen was introduced, since day 5 of menstruation till onset of next menstruation. The patient was asked to follow the same regimen for six months. The sequence included Padmasana (Lotus Pose), Trikonasana (Triangle Pose), Sarvangasana (Shoulder Stand), Baddha Konasana (Bound Angle Pose) and Savasana (Corpse Pose). The yoga session lasted approximately 30-40 minutes, followed by pranayamam for 5 minutes and was practiced in a quiet, well-ventilated space, with emphasis on breath awareness and gentle transitions also advised food recommendations and sleep pattern.

## Assessment tools for symptom severity and quality of life in dysmenorrhea:

[1] WaLIDD Score [15] a tool that provides multidimensional assessment of Dysmenorrhea

[2] Dysmenorrhea Symptom Interference (DSI) Scale [16] is a validated patient-reported outcome measure.

## Results and Discussion:

Following the application of Thermal cauterization therapy, the patient reported substantial relief from her dysmenorrhoeic symptoms (First cycle itself). The patient was monitored over a six months period, during which she consistently reported reduced pain intensity, improved concentration and enhanced quality of life during menstruation.

The bar chart illustrates a comparative analysis of life quality parameters across three menstrual phases: off menses, previous cycle during menses and present cycle during menses. Across all ten domains-mood, social and leisure activities, enjoyment of life, concentration, work, daily activities, sleep and physical activity-the present cycle shows notable improvement over the previous cycle, though still not fully restored to off-menses levels. The present case study demonstrates the therapeutic potential of Thermal cauterization in alleviating the complex burden of primary dysmenorrhea. Pain reduction was evident from the initial menstrual cycle following the administration of Thermal cauterization therapy. The observe reduction in dysmenorrheic pain during the initial treatment cycle, prior to the introduction of adjunctive interventions such as yoga and dietary modifications. Underscores the primary therapeutic role of thermal cauterization. This temporal association suggests that the early alleviation of symptoms can be attributed predominantly to the effect of Thermal cauterization therapy. Based on the WaLIDD score (Figure 2 - see PDF), the patient demonstrated a clear and progressive reduction in dysmenorrheic pain intensity across successive menstrual cycles. The initial score of 12 in the pre-intervention cycle reflected severe pain with significant disruption to daily functioning. Following the administration of Thermal cauterization therapy, the score declined to 7 in Cycle I, indicating persistent but reduced severity. By Cycle II, the score dropped further to 4, marking the transition into moderate dysmenorrhea. In Cycle III, the score reached 2.5 and by Cycle VI, it stabilized at 2, both indicative of mild symptomatology. This descending mechanical phenomenon from severe to mild pain correlates impermanent with the initiation and continuation of Thermal cauterization therapy, suggesting its central role in pain modulation. The consistent decline across cycles highlights both the efficacy and reproducibility of the intervention, achieved without reliance on pharmacological agents. The upward shift in the gray bars of DSI (VI cycle) suggests that the intervention (Figure 3 - see PDF), with Thermal cauterization therapy, contributed to enhanced functional capacity, emotional stability and overall well-being during menstruation. The data supports the hypothesis that targeted Siddha therapy can mitigate the systemic disruptions typically associated with dysmenorrhea, enabling better participation in daily life and in this case the symptoms were improved from moderate to mild. The use of crystal salt and ginger as thermal buffers ensured safety and enhanced heat penetration without epidermal damage. In the context of primary dysmenorrhea, where heightened prostaglandin activity leads to uterine hyper contractility and ischemia, targeted stimulation at the umbilicus via Thermal cauterization may exert both local and systemic effects. Iacovides *et al.* (2015) highlight that women with dysmenorrhea often exhibit peripheral and central sensitization, with amplified nociceptive signaling even outside the menstrual phase [17]. By applying controlled heat at the umbilicus, Thermal cauterization may modulate these sensitized pathways, promoting vasodilation, interrupting pain transmission and restoring autonomic balance. Neuromodulation appears to exert systemic effects on autonomic regulation, suggesting that peripheral stimulation can restore autonomic balance and contribute to pain relief beyond the site of application [18]. Follow of a structured yoga regimen throughout the rest of the menstrual cycle, played a complementary role in sustaining symptom relief and further amplified therapeutic outcomes. Yoga also modulates cortisol levels, activates the parasympathetic nervous system and promotes pelvic muscle relaxation, making it a valuable adjunct in dysmenorrhea management [19]. While single-subject designs limit generalizability, the reproducibility of the protocol, alignment with both traditional and biomedical frameworks and measurable outcomes support its potential for broader clinical application. Future studies with larger cohorts and controlled designs are warranted to validate efficacy, optimize timing and dosage and explore long-term benefits.

## Conclusion:

The therapeutic efficacy of Thermal cauterization as a primary nonpharmacological intervention in the pain management of dysmenorrhea is reported. Early and sustained reductions in pain intensity, duration and functional improvement were observed. The intervention was well-tolerated, reproducible and aligned with Siddha principles. Thus, we show the effect of Thermal cauterization therapy as a standalone modality in menstrual pain management and warrant further investigation through controlled clinical studies.

## Advancement to knowledge:

The Siddha system of medicine contributes to the advancement of pain management through unique external therapeutic approaches such as thermal cauterization. As a noninvasive procedure, thermal cauterization offers a distinctive approach by rapidly reducing inflammation and alleviating pain within a short duration. This expands the scientific understanding of integrative pain management by demonstrating how traditional modalities can achieve clinically relevant outcomes without invasive techniques. By documenting its efficacy and mechanism, such research not only validates Siddha practices in contemporary contexts but also enriches the global discourse on safe, culturally resonant and effective alternatives for inflammation control and pain relief.
